# Foreign Bodies Simulating a Congenital Palatal Fistula and Vascular Anomaly

**DOI:** 10.1155/2015/387092

**Published:** 2015-05-14

**Authors:** Mekonen Eshete, Fikre Abate, Taye Hailu, Mulualem Gessesse, Azeez Butali

**Affiliations:** ^1^Cleft Lip and Palate Program, Yekatit 12 Hospital Medical College, Addis Ababa, Ethiopia; ^2^Faculty of Health Sciences, School of Medicine, AAU, Ethiopia; ^3^Yekatit 12 Hospital Medical College, Addis Ababa, Ethiopia; ^4^Department of Oral Pathology, Radiology and Medicine and Dows Institute for Dental Research at the College of Dentistry, University of Iowa, ML2198, 500 Newton Road, Iowa City, IA 52242, USA

## Abstract

Foreign bodies embedded in the palate are uncommon findings and may occasionally mimic oral lesions. In the majority of the cases, foreign body embedment in the palate happens in infants and children who are unable to give history. Physical examination in the oral cavity of this group of patients in order to arrive at a definitive diagnosis is limited. We present two female infants with foreign bodies adherent to the hard palate. The first was ten months old and the second was 11 months old. In both cases the materials removed from the palate were plastic in nature (black or red in color and circular in shape). The first simulated a palatal fistula and the second a vascular anomaly.

## 1. Case Report

*Case I*. A ten-month-old female infant was brought to our unit by her mother following referral from a pediatrician with an impression of palatal fistula. The mother explained that the child is not feeding well, is choking recurrently, has epistaxis, and puts her finger into her mouth in an unusual manner. She noticed a black circular area on the palate of her child that looks like a hole. It is not known for how long the infant has had the above problem because the mother is a known HIV patient who was admitted to a hospital for a month. She explained that on discharge from a hospital she found her infant to have the above problems and took her to the nearest pediatrics unit from where the infant is referred to our hospital's pediatrics department. The infant is also on prophylaxis as an infant exposed to retrovirus infection. She was exclusively on breast-feeding for six months and now on formula feeding. At our hospital she was first seen at the pediatrics outpatient clinic and with the same impression the infant was referred to our cleft unit.

**Figure 1:**
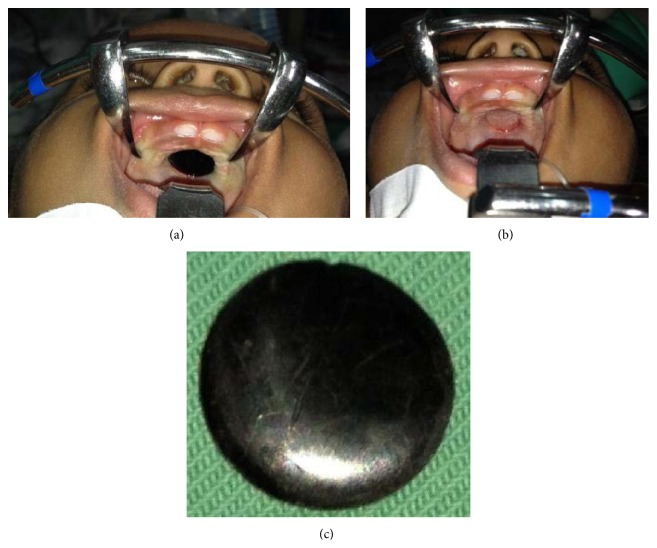
(a) The black foreign body embedded on the hard palate. (b) The foreign body was detached. (c) The extracted foreign body.

On presentation at our unit, the infant was not in any form of distress and her vital signs were stable. Proper intraoral examination without anesthesia was not possible because the infant was irritable and crying. She was subjected to examination under general anesthesia after a routine preparation. The examination revealed a circular black plastic foreign body embedded on the hard palate. It was covered with mucosa at its edge (Figure 1(a)). The foreign body was detached using artery forceps without making incisions (Figure 1(b)). There is an obvious indentation on the hard palate. The foreign body was a black colored concave plastic material with a diameter of 1.6 cm (Figure 1(c)).

*Case II*. An eleven-month-old female infant born to a primigravid mother at one of the provincial hospitals was brought by her mother to our center following referral from a pediatrician as a case of vascular malformation. Her current mother is a nurse working at the hospital where the infant was born. The biological mother was a teenager who disappeared from the hospital unnoticed after a day of the birth and the infant was cared by the hospital until her current mother legally adopted her.

**Figure 2:**
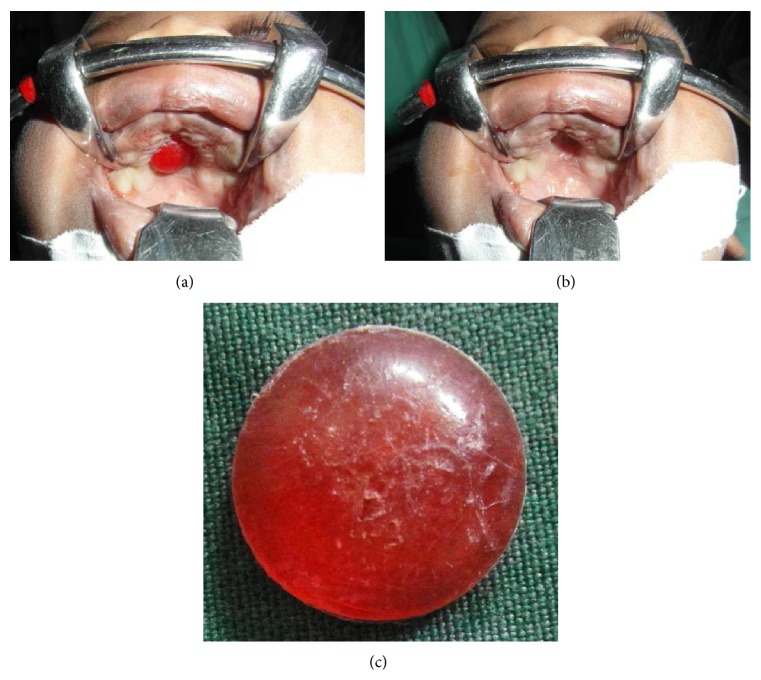
(a) The red foreign body deeply embedded in the hard palate. (b) Successful removal of the foreign body. The indentation on the hand palate is visible. (c) The extracted foreign body.

On physical examination at the plastic surgery unit, the infant was found to be comfortable with stable vital signs. Intraoral examination revealed a red colored foreign body deeply embedded in the hard palate (Figure 2(a)). Removal of the object at the clinic was attempted but unsuccessful. The child was subjected to general anesthesia and the foreign body was extracted using artery forceps. The indentation on the hard palate is visible (Figure 2(b)). The detached foreign body is found to be a concave red colored plastic material with a diameter of 1.6 cm (Figure 2(c)).

## 2. Discussion

Impaction of foreign bodies on the hard palate of infants and children is not a common finding. However, it should be considered in the differential diagnosis of palatal lesions in infants and children who are unable to give history and when physical examination is difficult.

We present two cases of foreign body impaction on the hard palate because these are rare and to our knowledge there is no report of foreign bodies embedded on the hard palate simulating congenital palatal fistula and vascular malformation. Arriving at accurate diagnosis was also a challenge. In the majority of the reported cases in the literature, foreign body impaction on the palate has been initially misdiagnosed with oral mucosa pathologies [1–4]. The most common referring diagnosis in the literature is palatal tumor [5–10]. The other presentations described in the literature are perforated palate, leukoplakia, torus palatinus, infection, and feeding difficulties [6, 10–14]. Fungal infection is one of the infectious conditions considered and led to initiation of unnecessary antifungal treatment [11].

There are some contributing factors for the misdiagnosis such as the poor infant attendance. In many places in developing world, infants are left to the care of their older brothers or sisters who are unable to give relevant information. This is most likely in our first patient who was also very irritated and crying and proper clinical examination without anesthesia was not possible. The brief and little investigative intraoral clinical examination could be another contributing factor for the misdiagnosis [15]. The true nature of these foreign bodies might also be obscured by mucosal molding to their edges that resulted in misdiagnosis and unnecessary attempted biopsy under general anesthesia [2]. Radiologic method of investigation like CT scan is not readily available in many developing countries and it is not affordable for the majority of the population. It might also be misleading [16] resulting in undue exposure to radiation. It is not easy to do proper intraoral physical examination in an infant who is upset and irritated from multiple manual examinations at different levels of the health system. Therefore examination under general anesthesia is mandatory to reach a definitive diagnosis and also to remove the foreign body while controlling airway to avoid aspiration.

The foreign bodies removed from the palate of both infants in our paper were circular concave plastic materials of the same size (16 mm in diameter) and shape but different in color, black and red. Other objects found impacted on the palate of infants and children were plastic teddy bear nose, a “press-on” nail, a plastic cap of a wardrobe puller, nut shell [2], screw cover [2, 8, 14], clothing button [2], pistachio nutshell [4], and billiard cue tip [17].

## 3. Conclusion

In our paper both the removed foreign bodies have the same size, plastic nature, and circular and concave shape. The concave shape might have contributed for the tight adherence of the foreign bodies to the palates. The similarity in shape, material, and size might indicate that they have a similar source. We recommend that easily detachable small objects should not be accessible to infants and children at the risk age. We also recommend considering foreign bodies while making the diagnosis of palatal lesions (conditions). Careful clinical examination should be made before making decisions on additional investigations such as biopsy and radiographic exploration. This will save cost and time and reduce or prevent patients from going through invasive procedures where they only need clinicians to be more conservative and patient.
